# Sarcopenic obesity and therapeutic outcomes in gastrointestinal surgical oncology: A meta-analysis

**DOI:** 10.3389/fnut.2022.921817

**Published:** 2022-07-22

**Authors:** Peiyu Wang, Shaodong Wang, Yi Ma, Haoran Li, Zheng Liu, Guihu Lin, Xiao Li, Fan Yang, Mantang Qiu

**Affiliations:** ^1^Department of Thoracic Surgery, Thoracic Oncology Institute, Peking University People’s Hospital, Beijing, China; ^2^Department of Thoracic Surgery, China Aerospace Science and Industry Corporation 731 Hospital, Beijing, China

**Keywords:** gastrointestinal cancer, postoperative complication, sarcopenic obesity, surgery, survival

## Abstract

**Background:**

Sarcopenic obesity (SO) has been indicated as a scientific and clinical priority in oncology. This meta-analysis aimed to investigate the impacts of preoperative SO on therapeutic outcomes in gastrointestinal surgical oncology.

**Methods:**

We searched the PubMed, EMBASE, and Cochrane Library databases through March 4th 2022 to identify cohort studies. Endpoints included postoperative complications and survival outcomes. Newcastle Ottawa Scale was used for quality assessment. Heterogeneity and publication bias were assessed. Subgroup analyses and sensitivity analyses were performed.

**Results:**

Twenty-six studies (8,729 participants) with moderate to good quality were included. The pooled average age was 65.6 [95% confidence interval (CI) 63.7–67.6] years. The significant heterogeneity in SO definition and diagnosis among studies was observed. Patients with SO showed increased incidences of total complications (odds ratio 1.30, 95% CI: 1.03–1.64, *P* = 0.030) and major complications (Clavien-Dindo grade ≥ IIIa, odds ratio 2.15, 95% CI: 1.39–3.32, *P* = 0.001). SO was particularly associated with the incidence of cardiac complications, leak complications, and organ/space infection. SO was also predictive of poor overall survival (hazard ratio 1.73, 95% CI: 1.46–2.06, *P* < 0.001) and disease-free survival (hazard ratio 1.41, 95% CI: 1.20–1.66, *P* < 0.001). SO defined as sarcopenia in combination with obesity showed greater association with adverse outcomes than that defined as an increased ratio of fat mass to muscle mass. A low prevalence rate of SO (< 10%) was associated with increased significance for adverse outcomes compared to the high prevalence rate of SO (> 20%).

**Conclusion:**

The SO was associated with increased complications and poor survival in gastrointestinal surgical oncology. Interventions aiming at SO have potentials to promote surgery benefits for patients with gastrointestinal cancers. The heterogeneity in SO definition and diagnosis among studies should be considered when interpreting these findings.

**Systematic Review Registration:**

[https://www.crd.york.ac.uk/prospero/display_record.php?RecordID=255286], identifier [CRD42021255286].

## Introduction

Sarcopenia, defined as the depletion of skeletal muscle mass and muscle strength (1), has been demonstrated to be associated with increased complications, poor prognosis, and reduced quality of life in cancer patients (2–4). Sarcopenia was frequently observed in patients with gastrointestinal cancers due to cancer factors (feeding difficulties, cancer cachexia, and anti-cancer therapies) and demography factors (old age, obesity, and inactivity) (4, 5). Obesity, indicated by high body mass index (BMI), abundant visceral fat area (VFA), and increased fat mass proportion, has been reported as a risk factor for perioperative morbidities but with differentiated impacts on long-term survival (6–8). With the combination of sarcopenia and obesity, sarcopenic obesity (SO) shows potential to affect therapeutic outcomes in cancer patients and is rising increased concerns in surgical oncology (9, 10). The European Society for Clinical Nutrition (ESPEN) and Metabolism and the European Association for the Study of Obesity (EASO) have recognized and indicated sarcopenic obesity as a scientific and clinical priority (11).

The hidden muscle wasting in SO indicated reduced peripheral protein preservation, which is mobilized during metabolic stress of major surgeries to support amino acids for the immune system, liver and gut (12, 13). Obesity is believed to increase anesthetic risk and surgical difficulty because of increased comorbidities and abundant visceral fat (6, 11). We hypothesized that SO detrimentally impact perioperative and survival outcomes in gastrointestinal surgical oncology. A number of studies have investigated these issues in gastrointestinal cancers, but the outcomes are highly controversial (14–39). The varied definition and diagnosis cut-offs of SO leaded to wide variations in prevalence rates, impeding the interpretation of published findings (14–39). Several reviews have described these issues (9, 10, 40), however, quantitative analyses have not been conducted. As the number of original studies increased significantly in the past few years (14–27), the controversies regarding the impacts of SO on therapeutic outcomes in gastrointestinal surgery become much more prominent. The varied SO definition and diagnosis cut-offs also warrant further investigation in subgroup analyses (40). A meta-analysis is thus required and appropriate to solve these issues.

Against these backgrounds, the present meta-analysis aimed to clarify the impacts of SO on therapeutic outcomes in gastrointestinal surgical oncology. The existing SO definitions and diagnosis criteria were assessed and compared regarding their clinical significance.

## Methods

This meta-analysis was preregistered with the PROSPERO International Prospective Register for Systemic Reviews (Registration no. CRD42021255286) and reported in line with PRISMA (Preferred Reporting Items for Systematic Reviews and Meta-Analyses) (41) and AMSTAR (Assessing the methodological quality of systematic reviews) Guidelines (42).

### Data sources and searches

Two reviewers independently searched the PubMed, EMBASE, and Cochrane Library databases (title and abstract) for papers published through September 10th 2021. The search strategy combined relevant search terms, such as “cancer or tumor or carcinoma or malignancy,” “sarcopenia or sarcopenic or myopenia or myopenic,” “obesity or obese or adiposity or adipose,” and “surgery or surgical or operation or operative or resection” (Supplementary Table 1). After September 10th 2021, the literature update was performed manually on a weekly basis until March 4th 2022. References of the retrieved articles were manually reviewed to identify additional studies. An advisory group consisting of three senior authors was established to solve any disagreement.

### Study selection

The inclusion criteria were as follows: (1) study population: patients with gastrointestinal cancer treated by radical surgery; (2) indicator: patients with SO (since no unique definition exists for SO, each study definition was applied); (3) comparison: patients without SO (NSO) or non-sarcopenic non-obesity (NN) patients; (4) outcomes: overall complication, major complication, overall survival, disease-free survival, and other surgical outcomes; (5) study type: prospective and retrospective cohort studies. The exclusion criteria included (1) enrollment of non-gastrointestinal cancers; (2) lack of interested outcomes; (3) narrative reviews, case reports, comments, editorials, or corresponding letters; (3) overlapping studies; and (4) non-English literature. Two authors independently reviewed the titles and abstracts to screen possibly eligible articles for full-text review. The reasons for exclusion in the full-text review were recorded in detail. During these processes, any disagreement was resolved by the adversary group.

### Data extraction

The Cochrane Good Practice data extraction template was used to establish a standardized form for data extraction (43). For each cohort study, we extracted data on study design, year of publication, sample, patient characteristics (age, sex, and country), tumor site, type of surgery, SO (definition, diagnosis, and prevalence), and surgical outcomes. Data from multivariable analyses were preferentially extracted and used than those from univariable analyses. Calculation and conversion of continuous data were performed based on established methods (44, 45). Estimated survival data were extracted according to Williamson et al.’s (46) and Parmar and Stewart (47) methods. Data were extracted by two authors and compared with one another, the discrepancies were resolved by checking the original articles.

### Quality assessment

Two reviewers independently assessed the quality of the included studies according to the Newcastle Ottawa Scale (NOS) for cohort studies (48). The NOS includes six aspects, eight scoring points, and a total score of 9 points. A score of 5 or below was considered low quality, a score of 6 or 7 was considered moderate quality, and a score of 8 or 9 was considered high quality. The quality of the quantitatively pooled outcomes was assessed using the Grading of Recommendation Assessment, Development, and Evaluation (GRADE) system (49). Outcomes were allocated a score based on risk of bias, inconsistency, indirectness, imprecision, and publication bias, yielding an objective score with a GRADE rating ranging from 1 (very low quality) to 4 (high quality).

### Endpoints

Primary endpoints were overall complications, major complications, overall survival, and disease-free survival. Overall complications were defined as Clavien-Dindo grade ≥ I while major complications were defined as Clavien-Dindo grade ≥ IIIa (50). Overall survival was calculated from the time of surgery to the time of death from any causes. Disease-free survival was calculated from the time from surgery to the first recurrence of index cancer or to all-cause death. Secondary endpoints included operative time, blood loss, specific complications, postoperative hospital stay, unplanned readmission, and 30-days/in-hospital mortality. The impacts of SO definition and prevalence on endpoints analysis were particularly investigated.

### Statistical analysis

The applied summary statistics included the effect sizes with 95% confidence intervals (CIs) for prevalence rates, the odds ratio (ORs) with 95% CI for categorical data, the weighted mean difference (WMD) with 95% CI for continuous data, and the hazard ratio (HR) with 95% CI for survival data. The Mantel-Haenszel method and the inverse variance method were appropriately applied. The between-study heterogeneity was estimated with Cochran’s Q statistic using chi-square and *I*^2^ statistics. *I*^2^-values of 0–25, 25–50, and > 50% indicated low, moderate, and high heterogeneity, respectively. Fixed effect models were used for meta-analyses with low to moderate heterogeneity. Subgroup analyses regarding cancer types, SO definition, and SO prevalence were conducted to identify potential sources of significant heterogeneity, or a random effect model was adopted. We assessed publication bias using funnel plots and Egger’s test. We also conducted sensitivity analysis by omitting one study at a time to examine the influence of each study on the pooled outcomes. A two-tailed *P*-value < 0.05 was considered statistically significant. All analyses were conducted with STATA version 16 software (Stata Corporation, College Station, TX, United States).

## Results

### Study selection and characteristics

**FIGURE 1:**
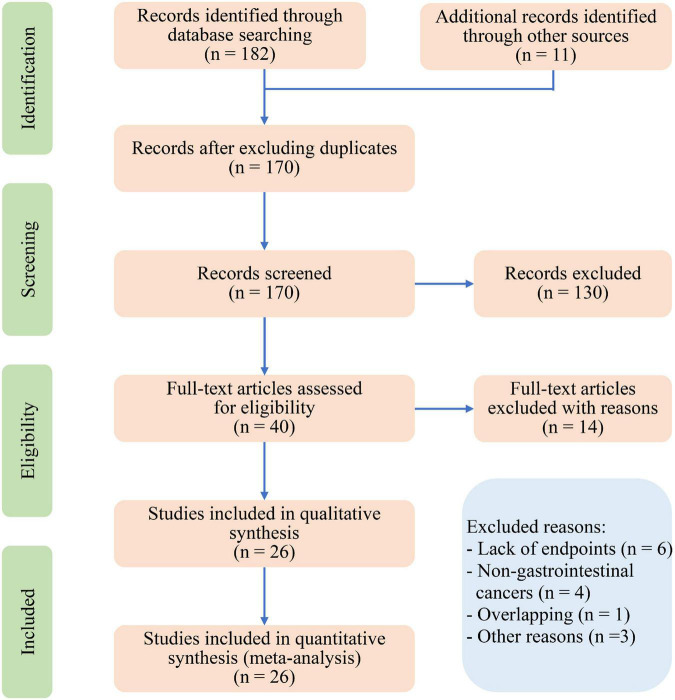
Preferred reporting items for systematic review and meta-analysis (PRISMA) flow diagram for study selection.

The primary literature review identified 170 relevant papers (Figure 1). The review of titles and abstracts excluded 130 papers, leaving 40 articles for full review. Twenty-six studies with a total of 8,729 participants were finally included after evaluating the full papers (Table 1) (14–39). A list of excluded studies with reasons is shown in Supplementary Table 2. Among the included studies, twenty-four studies were retrospective, and two studies were prospective. The diagnoses included gastric cancer (*n* = 1,980) (14, 16, 17, 28, 34), esophageal cancer (*n* = 296) (18, 20, 36), hepatic cancer (*n* = 535) (23, 24), pancreatic cancer (*n* = 1,160) (15, 25, 26, 31–33), colorectal cancer (*n* = 4,328), (19, 21, 22, 27, 29, 30, 35, 37) and colorectal liver metastases (*n* = 430) (38, 39). The pooled average age of 5,784 patients was 65.6 (95% CI: 63.7–67.6) years (14–16, 18–21, 23–27, 30–33, 35–39).

**TABLE 1:** Study characteristics.

Studies	Cancer type	Design	Country	Sample	Male (%)	Age^a^	Sarcopenic obesity		Major endpoints
							Diagnosis method	Prevalence (%)	
Rodrigues et al. (14)	Gastric	Retro	Spain	198	57.6	73.5	SMI + VFA	55 (27.8)	Complication; OS; DFS
Peng et al. (15)	Pancreatic	Retro	China	116	58.6	*66.2*	SMI + VFA/TAMA	3 (2.6)	OS, DFS
Olmez et al. (16)	Gastric	Retro	Turkey	149	65.8	*59.3*	SMI + BMI	10 (6.7)	Complication
Kim et al. (17)	Gastric	Retro	Korea	840	62.6	*60.4*	SMI + VFA	48 (5.7)	OS
Fehrenbach et al. (18)	Esophageal	Retro	Germany	85	88.2	*64.3*	SMI + BMI	7 (8.2)	Complication; OS; DFS
Pedrazzani et al. (19)	Colorectal	Retro	Italy	261	56.7	*67.9*	VFA/TAMA	87 (33.3)	Complication
Onishi et al. (20)	Esophageal	Retro	Japan	91	80.2	*74.1*	SMI + VFA	31 (34.1)	Complication; OS
Han et al. (21)	Rectal	Retro	Korea	1,384	64.2	*59.0*	VFA/TAMA	307 (22.2)	OS; DFS
Giani et al. (22)	Rectal	Retro	Italy	173	64.2	NR	VFA/TAMA	43 (24.9)	Complication
Kroh et al. (23)	Hepatic	Retro	Germany	70	70.0	*67.7*	SMI + fat mass %	21 (30.0)	Complication; OS
Kobayashi et al. (24)	Hepatic	Retro	Japan	465	78.9	*67.6*	SMI + VFA	31 (6.7)	Complication; OS; DFS
Jang et al. (25)	Pancreatic	Retro	Korea	284	57.4	*62.6*	VFA/TAMA	84 (29.6)	Complication
Gruber et al. (26)	Pancreatic	Retro	Austria	133	51.1	65	SMI + BMI	34 (25.6)	Complication; OS; DFS
Berkel et al. (27)	Rectal	Retro	Netherlands	99	53.5	66	SMI + BMI	NR	Complication; OS
Zhang et al. (28)	Gastric	Pro	China	636	75.2	NR	AWGS + VFA/BMI	39 (6.1)	Complication
Martin et al. (29)	Colorectal	Retro	Canada, United Kingdom	1,139	60.4	NR	SMI + VFAI	47 (4.1)	Complication
Chen et al. (30)	Colorectal	Pro	China	376	60.6	*64.3*	AWGS + VFA	41 (10.9)	Complication
Okumura et al. (31)	Pancreatic	Retro	Greece	301	55.8	68	SMI + VFA	57 (18.9)	Complication; OS; DFS
Sandini et al. (32)	Pancreatic	Retro	Italy	124	50.8	72	VFA/TAMA	61 (49.2)	Complication
Pecorelli et al. (33)	Pancreatic	Retro	Italy	202	53.5	*66⋅8*	VFA/TAMA	NR	Complication; OS
Nishigori et al. (34)	Gastric	Retro	China	157	65.6	*NR*	SMI + VFA	45 (28.7)	Complication
Malietzis et al. (35)	Colorectal	Retro	United Kingdom	805	58.6	69	SMI + BMI	80 (9.9)	Complication; OS; DFS
Grotenhuis et al. (36)	Esophageal	Retro	Netherlands	120	73.3	62	SMI + BMI	29 (24.2)	Complication; OS; DFS
Boer et al. (37)	Colon	Retro	Netherlands	91	53.8	*71.3*	SMI + BMI	26 (28.6)	Complication; OS
Lodewick et al. (38)	CLM	Retro	Netherlands	171	60.8	64	SMI + fat mass %	49 (28.7)	Complication; OS; DFS
Peng et al. (39)	CLM	Retro	United States	259	59.8	*58*	TPA + BMI	5 (1.9)	Complication; OS; DFS

^a^Age is shown as median value or mean value of years.

AWGS, Asian Working Group for Sarcopenia; BMI, body mass index; CLM, colorectal liver metastasis; DFS, disease-free survival; NR, not reported; OS, overall survival; SMI, skeletal muscle index; TAMA, total abdominal muscle area; TPA, total psoas muscle area; VFA, visceral fat area.

### Quality assessment of individual studies

Quality assessments by Newcastle-Ottawa Scale are summarized in Supplementary Table 3. Of the 26 studies, nine studies (14, 19, 20, 23, 24, 26, 28, 30, 38) were of high quality and seventeen studies (15–18, 21, 22, 25, 27, 29, 31–37, 39) were of moderate quality.

### Definition and prevalence of sarcopenic obesity

The diversity of SO definitions and prevalence is summarized in Table 2. Twenty studies defined SO as sarcopenia in combination with obesity, while six studies defined SO as an increased ratio of fat mass to skeletal muscle mass. Sarcopenia was mostly diagnosed as a low skeletal muscle index (SMI), which was calculated by normalizing the total abdominal muscle area at vertebral level L3 in square centimeters by the height in square meters. Two prospective studies (28, 30) assessed sarcopenia according to the Asian Working Group for Sarcopenia criteria (1). Obesity was mostly diagnosed as high BMI (> 30 or 25 kg/m^2^) or abundant VFA (mostly > 100 cm^2^). The specific cutoffs for diagnosing sarcopenia and obesity were inconsistent across studies, leading to a varied prevalence rate of SO ranging between 1.9 and 30.0%. Generally, the rigorous diagnostic cutoffs led to low prevalence rates of SO. Six studies defined SO as an increased ratio of fat mass to muscle mass and reported a high prevalence rate of SO ranging from 22.2 to 49.2% (19, 21, 22, 25, 32, 33). Based on the distribution of SO prevalence rates (Supplementary Figure 1), we divided the included studies into groups of high prevalence (> 20%) and low prevalence (< 10%). The subgroup analysis demonstrated that the prevalence rate of SO was 28.4% (95% CI: 24.9–31.9) in the high prevalence group and 6.1% (95% CI: 4.3–7.8) in the low prevalence group without publication bias (Supplementary Figure 2).

**TABLE 2:** Panels of sarcopenic obesity definitions, criteria, and prevalence.

Panels	Sarcopenia			Obesity			Prevalence	References
	Parameter	Tool	Cut off (cm/m^2^)	Parameter	Tool	Cut off		
1.1	SMI	CT-L3	M 52.4; F 38.5	BMI	W/H^2^	≥30 kg/m^2^	6.7%	(16)
							8.2%	(18)
							9.9%	(35)
1.2	SMI	CT-L3	M 52.4; F 38.5	BMI	W/H^2^	≥ 25 kg/m^2^	25.6%	(26)
							24.2%	(36)
1.3	SMI	CT-L3	Median	BMI	W/H^2^	≥ 25 kg/m^2^	NR	(27)
							28.6%	(37)
1.4	TPA	CT-L3	500 mm^2^/m^2^	BMI	W/H^2^	≥ 30 kg/m^2^	1.9%	(39)
2.1	SMI	CT-L3	M 52.4; F 38.5	VFA	CT-L3	M 163.8; F 80.1 cm^2^	27.8%	(14)
				VFA	CT-L3	M/F > 100 cm^2^	28.7%	(34)
2.2	SMI	CT-L3	M 49.0; F 31.0	VFA	CT-L3	M/F > 100 cm^2^	5.7%	(17)
	SMI	CT-L3	M 42.0; F 38.0				34.1%	(20)
	SMI	CT-L3	M 40.3; F 30.9				6.7%	(24)
	SMI	CT-L3	M 47.1; F 36.6				18.9%	(31)
3	SMI	CT-L3	M 43.0/53.0 F 41.0	Fat mass%	CT-L3	Top two quintiles	30.0%	(23)
				Fat mass%	CT-L3	M 35.7; F 44.4	28.7%	(38)
5	SMI	CT-L3	M 42.2; F 33.9	VFA/TAMA	CT-L3	≥ 2	2.6%	(15)
	SMI	CT-L3	z-score < –0.5	VFAI	CT-L3	z-score > 0.5	4.1%	(29)
4	AWGS	CT-L3 SMI	M 40.8; F 34.9	VFA	CT-L3	M:132.6; F: 91.5 cm^2^	6.1%	(28)
		HGS	M 26; F 18 kg	BMI	W/H^2^	M 24.1; F 23.1 kg/m^2^		
		6-m gait speed	0.8 m/s	VFA	CT-L3	M 130; F 90 cm^2^	10.9%	(30)
6	VFA/TAMA based on CT-L3 with the cutoff of > 3.2						22.2%	(21)
							29.6%	(25)
							NR	(33)
	VFA/TAMA based on CT-L3 with gender-specific cutoffs of the second tertile						33.3%	(19)
	VFA/TAMA based on CT-L3 with gender-specific cutoffs of the fourth quartile						24.9%	(22)
	VFA/TAMA based on CT-L3 with gender-specific cutoffs: M 2.8; F 2.4						49.2%	(32)

AWGS, Asian Working Group for Sarcopenia; BMI, body mass index; CT, computed tomography; F, female; HGS, handgrip strength; H, height; M, male; NR, not reported; SMI, skeletal muscle index; TAMA, total abdominal muscle area; TPA, total psoas muscle area; VFA, visceral fat area; W, weight.

### Clinical characteristics of sarcopenic obesity patients

As shown in Supplementary Table 4, patients with SO were older than NN patients (WMD 8.65, 95% CI: 6.24–11.05, *P* < 0.001), while the difference between patients with and without SO was not significant (WMD 4.02, 95% CI: –0.08 to 8.13, *P* = 0.055). Patients with SO showed increased American Society of Anesthesiology grades (3–4) than both NN and NSO patients without heterogeneity (OR 2.99, 95% CI: 1.99–4.49, *P* < 0.001; OR 1.64, 95% CI: 1.26–2.15, *P* < 0.001, respectively). However, no significant difference in sex (male) or cancer stage (III–IV) was detected between SO and NN/NSO patients.

### Primary outcomes

GRADE evidence profiles of quantitative analyses for primary endpoints were summarized in Table 3. The assessed quality was mostly moderate.

**TABLE 3:** GRADE evidence profile: meta-analyses of sarcopenic obesity and primary endpoints.

Outcomes	No. of studies	Certainty assessment					Effect	Quality	Forest plots
		limitations	Inconsistency	Indirectness	Imprecision	Publication bias	HR (95% CI)		
**Overall complications**									
SO vs. NN	4 (14, 24, 30, 36)	Serious	Serious	Not serious	Not serious	Undetected	1.97 (1.28–3.02)	⊕⊕○○ (Low)	Figure 1A
SO vs. NSO	10 (14, 18, 19, 22–24, 30, 36–38)	Serious	Not serious	Not serious	Not serious	Undetected	1.30 (1.03–1.64)	⊕⊕⊕○ (Moderate)	Figure 1A
**Major complications**									
SO vs. NN	4 (14, 24, 28, 29)	Serious	Serious	Not serious	Not serious	Undetected	2.12 (1.36–3.31)	⊕⊕○○ (Low)	Figure 1B
SO vs. NSO	15 (14, 19, 20, 23–28, 31, 32, 35, 37–39)	Serious	Serious	Not serious	Not serious	Undetected	1.96 (1.56–2.47)	⊕⊕○○ (Low)	Figure 1B
**Overall survival**									
SO vs. NN/NSO	11 (14, 15, 17, 18, 20, 21, 23, 24, 31, 33, 35)	Serious	Not serious	Not serious	Not serious	Undetected	1.73 (1.46–2.06)	⊕⊕⊕○ (Moderate)	Figure 1C
SO vs. NSO	9 (15, 17, 18, 20, 21, 23, 31, 33, 35)	Serious	Not serious	Not serious	Not serious	Undetected	1.69 (1.41–2.03)	⊕⊕⊕○ (Moderate)	Figure 1C
SO vs. NN/NSO*^a^*	7 (14, 15, 20, 21, 24, 26, 31)	Not serious	Not serious	Not serious	Not serious	Undetected	1.62 (1.35–1.95)	⊕⊕⊕⊕ (High)	No plot
**Disease-free survival**									
SO vs. NN/NSO	8 (14, 15, 18, 21, 24, 26, 31, 35)	Serious	Not serious	Not serious	Not serious	Undetected	1.41 (1.20–1.66)	⊕⊕⊕○ (Moderate)	Figure 1D
SO vs. NSO	6 (15, 18, 21, 26, 31, 35)	Serious	Not serious	Not serious	Not serious	Undetected	1.33 (1.11–1.59)	⊕⊕⊕○ (Moderate)	Figure 1D
SO vs. NN/NSO^a^	3 (14, 24, 31)	Not serious	Not serious	Not serious	Not serious	Undetected	1.87 (1.44–2.43)	⊕⊕⊕⊕ (High)	No plot

^a^The outcome of meta-analyses were based on extracted data from multivariable analyses, while other outcomes of meta-analyses were based on data extracted from univariable analyses. GRADE, Grading of Recommendation Assessment, Development, and Evaluation system; CI, confidence interval; HR, hazard rate; NN, non-sarcopenic non-obesity; NSO, patients without sarcopenic obesity; SO, sarcopenic obesity.

### Total complications

**FIGURE 2:**
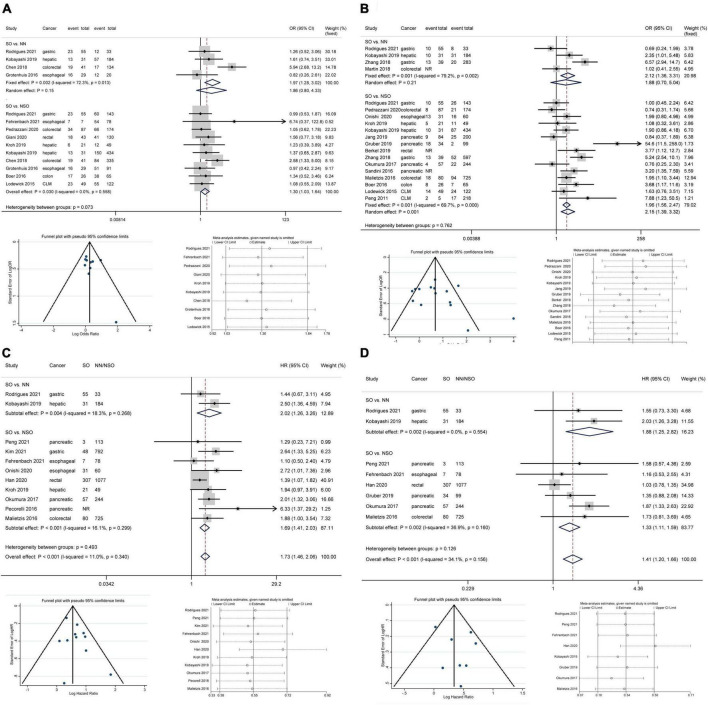
Forest plots of meta-analyses for sarcopenic obesity and primary outcomes. The funnel plots and sensitivity analyses of SO vs. NSO are provided. **(A)** Total complications; **(B)** major complications; **(C)** overall survival; **(D)** disease-free survival. CI, confidence interval; CLM, colorectal liver metastasis; HR, hazard ratio; NN, non-sarcopenic non-obesity; NR, not reported; NSO, patients without sarcopenic obesity; OR, odds ratio; SO, sarcopenic obesity.

**FIGURE 3:**
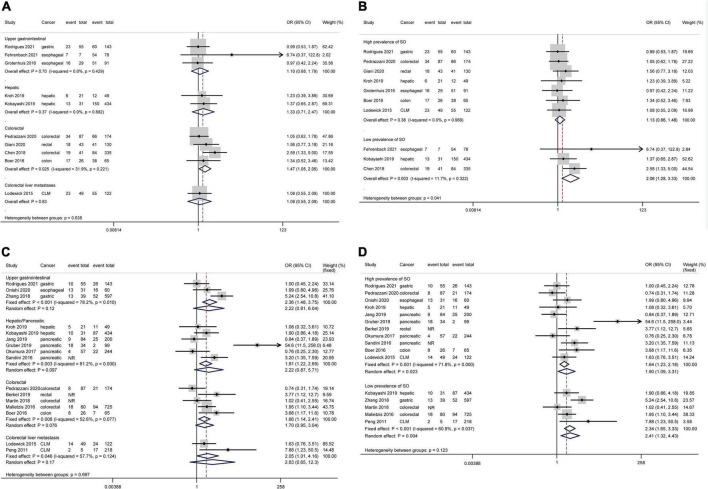
Forest plots of subgroup analyses for sarcopenic obesity and postoperative complications stratified by cancer type and sarcopenic obesity prevalence. Total complications: **(A,B)** Major complications: **(C,D)**. CI, confidence interval; CLM, colorectal liver metastasis; OR, odds ratio; SO, sarcopenic obesity.

Based on 10 studies comprising 2010 patients and 389 complications, patients with SO showed an increased risk of total complications than patients without SO without heterogeneity or publication bias (Figure 2A, OR 1.30, 95% CI: 1.03–1.64, *P* = 0.030; *I*^2^ = 0%). The pooled analysis of 4 studies demonstrated an increased risk of total complications in patients with SO compared with NN patients using a fixed effect model, but the difference was not significant in the random effect model. The subgroup analysis regarding cancer types (Figure 3A) only demonstrated the SO as a risk factor for total complications in colorectal cancers, but no significant between-group heterogeneity was detected (*P* = 0.64). The subgroup analysis regarding the SO prevalence rate (Figure 3B) demonstrated that SO was a risk factor for total complications in the low prevalence group but not in the high prevalence group. The subgroup analyses regarding SO definitions (Supplementary Figure 3A) demonstrated that SO defined as sarcopenia in combination with obesity was a risk factor for total complications while SO defined as increased ratio of fat mass to muscle mass was not significantly associated with the outcome. The quality of these subgroup analyses was moderate according to the GRADE system (Table 3).

### Major complications

All included studies defined major complications as Clavien-Dindo grade ≥ IIIa except for the study by Gruber et al. (26), which adopted the criterion of Clavien-Dindo grade ≥ IIIb. Based on 15 studies comprising 3,952 patients and 660 major complications, SO patients showed an increased risk of major complications compared to NSO patients with significant heterogeneity but no publication bias (Figure 2B, OR 2.15, 95% CI: 1.39–3.32, *P* = 0.001; *I*^2^ = 69.7%). The pooled analysis of 4 studies demonstrated an increased risk of major complications in patients with SO compared with NN patients using a fixed effect model, but the difference was not significant in the random effect model. The subgroup analysis regarding cancer types (Figure 3C) commonly demonstrated SO as a risk factor for major complications using fixed effect models, while these differences were not significant in random effect models. The subgroup analysis regarding the SO prevalence rate (Figure 3D) demonstrated that SO was a risk factor for major complications in both the low and the high prevalence groups regardless of the effect models adopted. Additionally, the subgroup analyses regarding SO definitions (Supplementary Figure 3B) demonstrated the SO defined as sarcopenia in combination with obesity rather than the increased ratio of fat mass to muscle mass as a risk factor for major complications. However, the GRADE quality of these subgroup analyses was mostly low because of the significant heterogeneity (Table 3).

### Overall survival

**FIGURE 4:**
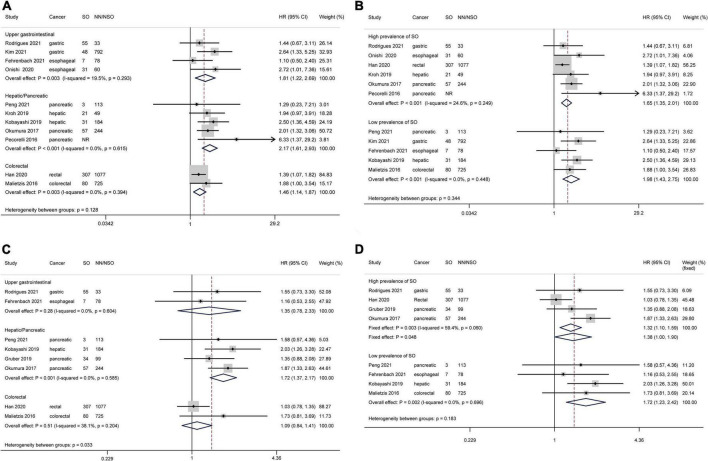
Forest plots of subgroup analyses for sarcopenic obesity and survival outcomes stratified by cancer type and sarcopenic obesity prevalence. Overall survival: **(A,B)** Disease-free survival: **(C,D)** CI, confidence interval; HR, hazard ratio; NN, non-sarcopenia non-obesity; NR, not reported; NSO, patients without sarcopenic obesity; SO, sarcopenic obesity.

Considering the differences in surgical radicality between primary gastrointestinal cancers and colorectal liver metastases, the latter were excluded from survival analyses. Based on 11 studies comprising 4,197 patients, patients with SO were associated with a shorter OS than NN/NSO patients without significant heterogeneity or publication bias (Figure 2C, HR 1.73, 95% CI: 1.46–2.06, *P* < 0.001; *I*^2^ = 0%). The heterogeneity between groups was not significant (*P* = 0.49). The subgroup analysis regarding cancer types (Figure 4A) demonstrated that SO was an adverse prognostic factor for OS across cancer groups. The subgroup analysis regarding SO prevalence (Figure 4B) confirmed the association between SO and shorter OS in both the low and the high prevalence groups. In addition, SO defined as sarcopenia combined with obesity showed a better association with poor OS than that defined as increased ratio of fat mass to muscle mass (Supplementary Figure 3C). Meta-analysis of multivariable data also demonstrated the predictive value of SO for poor OS (HR 1.62, 95% CI: 1.35–1.95, *P* < 0.001; *I*^2^ = 0%; 7 studies with 2,328 participants) (14, 15, 20, 21, 24, 26, 31). The GRADE quality of these subgroup analyses was moderate to high (Table 3).

### Disease-free survival

Based on 8 studies comprising 3,127 patients, patient with SO showed a shorter DFS than NN/NSO patients, with moderate heterogeneity but no publication bias (Figure 2D, HR 1.41, 95% CI: 1.20–1.66, *P* < 0.001; *I*^2^ = 34.1%). The heterogeneity between groups was not significant (*P* = 0.13). The subgroup analysis regarding cancer types (Figure 4C) demonstrated that SO was an adverse prognostic factor for DFS in hepatic/pancreatic cancers with significant between-group heterogeneity (*P* = 0.033). Regarding the subgroup analysis of SO prevalence (Figure 4D), SO was associated with shorter DFS in both the low and the high prevalence groups. The SO defined as sarcopenia in combination with obesity showed better predictive value for DFS than that defined as the increased ratio of fat mass to muscle mass (Supplementary Figure 3D). SO was still associated with poor DFS in the pooled analysis of multivariable data (HR 1.87, 95% CI: 1.44–2.43, *P* < 0.001; *I*^2^ = 0%; 3 studies with 604 participants) (14, 24, 31). The quality of these subgroup analyses was moderate to high according to the GRADE system (Table 3).

### Secondary outcomes

Meta-analyses for secondary outcomes and GRADE quality assessments are summarized in Table 4. No significant difference in operative time or blood loss was detected between SO and NN/NSO patients. SO was demonstrated to be associated with the incidence of pulmonary complications using a fixed effect model (OR 2.26, 95% CI: 1.43–3.59, *P* < 0.001; *I*^2^ = 55.6%), but the difference was not significant in the random effect model. SO was associated with the incidence of cardiac complications in a small pooled sample. Based on 11 studies comprising 2,701 patients and 293 leak complications, SO was demonstrated as a risk factor for leak complications without significant heterogeneity or publication bias; however, the differences were not significant in subgroup analyses of anastomotic leakage and pancreatic fistula. Patients with SO also showed an increased risk of organ/space infection compared to NN/NSO patients. Furthermore, the pooled analysis of 4 studies showed a higher risk of unplanned readmission in patients with SO than in NN/NSO patients. Regarding perioperative mortality, the subgroup analysis demonstrated the SO as a risk factor for perioperative mortality in the low prevalence group but not in the high prevalence group. The GRADE quality of these analyses was mostly moderate (Table 4).

**TABLE 4:** Meta-analyses of second endpoints in sarcopenia obesity patients.

Parameter	Comparisons	Study and sample	Meta-analysis		Heterogeneity^a^		Publication bias		Sensitivity analysis	GRADE quality
			OR/*WMD* (95% CI)	*P*-value	*I*^2^ (%)	*P*	Funnel plot	Egger’s test		
Intraoperative parameters										
Operative time, min	SO vs. NN/NSO	429 (19, 20, 34)	*10.1 (–23.3 to 43.5)*	0.55	59.4	0.085	Negative	0.39	Negative	Low
Blood loss, ml	SO vs. NN/NSO	553 (19, 24, 34)	*5.93 (–12.5 to 24.4)*	0.53	0	0.56	Negative	0.82	Positive	Moderate
Specific complications										
Pulmonary complications	SO vs. NSO	1,007 (18–20, 23, 30, 32)	2.34 (0.99–5.50)	0.051	55.6	0.046	Negative	0.38	Negative	Low
Cardiac complications	SO vs. NSO	831 (19, 23, 30, 32)	3.81 (1.93–7.53)	<0.001	0	0.98	Negative	0.084	Negative	Moderate
Leak complications	SO vs. NSO	2,701 (18, 19, 22, 23, 25, 26, 30–32, 34, 35)	1.50 (1.11–2.04)	0.009	13.1	0.32	Negative	0.17	Negative	Moderate
Anastomotic leakage	SO vs. NSO	1,769 (18, 19, 22, 30, 34, 35)	1.51 (0.90–2.53)	0.12	0	0.91	Negative	0.032	Negative	Moderate
Pancreatic fistula	SO vs. NSO	840 (25, 26, 31, 32)	1.38 (0.67–2.83)	0.38	65.2	0.035	Negative	0.033	Negative	Low
Organ/space infection	SO vs. NN	328 (16, 30, 34)	5.23 (1.99–13.7)	0.001	0	0.90	Negative	058	Negative	Moderate
	SO vs. NSO	1,331 (16, 19, 20, 22, 30, 32, 34)	1.80 (1.23–2.63)	0.003	31.7	0.19	Negative	0.080	Negative	Moderate
Postoperative hospital stay	SO vs. NN/NSO	1,650 (19, 20, 28, 35, 38)	*1.74 (*–*0.71 to 4.19)*	0.17	68.3	0.013	Negative	0.20	Negative	Low
Readmission	SO vs. NN/NSO	1,175 (19, 29, 30, 38)	2.49 (1.40–4.45)	0.002	0	0.99	Negative	0.15	Negative	Moderate
30 d/in–hospital mortality	SO vs. NSO	2,391 (14, 19, 23, 28, 31, 35, 36)	2.27 (1.22–4.23)	0.010	62.6	0.013	Negative	0.65	Positive	Low
High prevalence of SO	SO vs. NSO	950 (14, 19, 23, 31, 36)	1.07 (0.46–2.46)	0.88	0	0.87	Negative	0.56	Negative	Moderate
Low prevalence of SO	SO vs. NSO	1,441 (28, 35)	18.4 (5.78–58.8)	<0.001	0	0.45	Negative	-	Negative	Moderate

^a^Once high heterogeneity was confirmed (I^2^ > 50% or P < 0.05), a random-effects model was adopted, otherwise, a fixed-effects model was used.

GRADE, Grading of Recommendation Assessment, Development, and Evaluation system; CI, confidence interval; NN, non-sarcopenia, non-obesity; NSO, patients without sarcopenic obesity; OR, odds ratio; SO, sarcopenic obesity; WMD, weighted mean difference.

## Discussion

This is the first meta-analysis investigating the impact of SO on the therapeutic outcomes of gastrointestinal cancer surgeries. Subgroup analyses were conducted to solve the heterogeneity caused by various SO definition and diagnosis cut-offs. Patients with SO showed increased risk of perioperative morbidities and compromised survival outcomes.

The detrimental impacts of SO on systematic therapeutic outcomes in cancer patients have been partially investigated (9, 51). SO was demonstrated to be associated with poor prognosis, chemotherapy toxicity, and reduced quality of life. Regarding its role in surgical oncology, an increasing number of studies have been published since 2020 (Table 1). Apart from gastrointestinal cancers, SO was also reported to be associated with perioperative complications and poor survival in the surgical treatment of lung cancer and endometrial cancer (52, 53). There are also reports of poor survival benefit in patients with SO who have underwent liver transplantation for hepatic cancers (54, 55). However, most of these published studies were retrospectively conducted in a single center with small samples. This meta-analysis confirmed the increased complications and poorer survival profiles in patients with SO than in NN/NSO patients. In particular, SO was associated with an increased risk of cardiac complications, leak complications, organ/space infection, unplanned readmission and perioperative mortality. SO defined with rigorous cutoffs led to prolonged hospital stay (18, 28–30). However, the imbalances of baseline characteristics between SO and NN/NSO patients should be considered when interpreting these findings. Advanced age and elevated American Society of Anesthesiology grade in patients with SO may be confounding factors. Several multivariable analyses have confirmed SO as an independent risk factor for postoperative complications (19, 25, 27, 28, 30, 32–34). The meta-analyses of multivariable survival data also confirmed the predictive value of SO for poor OS and DFS.

Mechanisms underlying the association between SO and adverse therapeutic outcomes have not been clarified. Patients with SO seems to carry adverse characteristics of both sarcopenia and obesity based on the mentioned evidence. Sarcopenia, mostly caused by the aging process, cancer consumption, and anti-cancer therapy (5, 56), is linked to advanced stage and poor outcomes in gastrointestinal cancers (2, 4, 57). Skeletal muscle serves as an important peripheral protein preservation mobilized during the perioperative period to provide amino acids supporting inflammatory reactions, immune responses, issue repair, and metabolic stress (3, 13). In addition, skeletal muscle has been increasingly confirmed as the potential central link between sarcopenia and immune senescence (58, 59). Myokines from skeletal muscle, such as interleukin (IL)-15, IL-17, and IL-6, modulate the proliferation and function of immune cells. Muscle wasting has also been demonstrated to be an independent and unfavorable prognostic factor in patients with advanced cancer receiving immune checkpoint inhibitors (60). These adverse characteristics may account for the increased complications and poor survival in SO patients in gastrointestinal surgical oncology. On the other hand, obesity is frequently accompanied by increased comorbidities represented by diabetes and cardiovascular diseases (11) and has been reported as a risk factor for cardiac complications and anastomotic leakage after major surgeries (6, 61). Adipose-mediated inflammation has been demonstrated to cause immune dysfunction in the obese population (62, 63). Intriguingly, obesity was reported to be associated with early recurrence but not overall mortality in gastrointestinal cancer patients (7). The obesity paradox, i.e., the U-shape relationship between mortality and BMI-defined adiposity (64), may impact the demonstrated characteristics of SO. In elderly adults with SO, obesity was demonstrated to have protective effects against the impaired functional status (65). The NN patients was thus the preferred comparator for revealing real characteristics of patients with SO. Collectively, sarcopenia and obesity may impact therapeutic outcomes in multidimensional and complex manners, and the hidden skeletal muscle wasting seems to be predominant in compromising survival benefits in patients with SO.

Recently, the ESPEN and EASO have launched consensus on definition and diagnostic criteria for SO (66). Diagnostic procedures initially include assessment of skeletal muscle function, followed by assessment of body composition for excess adiposity and low skeletal muscle mass. However, this consensus has not been generalized in surgical oncology. In this meta-analysis, regarding SO diagnosis (Tables 1, 2), the SO definition and diagnosis cut-offs instead of cancer types were demonstrated to be prominent factors influencing the reported prevalence rates. Regarding clinical significances, SO defined as sarcopenia in combination with obesity showed a greater association with postoperative complications and poor survival outcomes than that defined as an increased fat mass ratio to skeletal mass. The definition of sarcopenia in combination with obesity also conforms to the ESPEN-EASO consensus (66). Furthermore, the rigorous diagnosis cutoffs of SO lead to low prevalence but increased significance of adverse therapeutic outcomes. Correspondingly, studies (18, 28–30) that reported a low prevalence rate of SO commonly demonstrated SO as a risk factor for prolonged postoperative hospital stay, while studies (19, 20, 26, 38) that reported a high prevalence rate of SO demonstrated no significant association. All these findings indicate the importance of establishment and selection of diagnosis cut-offs for SO, which was not only a methodological problem but also impacted the clinical significance. Authoritative cut-offs for assessing obesity and sarcopenia can be found in ESPEN-EASO consensus (66), where the age-adjusted and gender-specific percentage of fat mass was recommended for diagnosing obesity and the percentage of skeletal muscle mass based on thresholds derived from healthy young population were recommended for diagnosing low skeletal muscle mass. Notably, the assessment of skeletal muscle function could be more important than that of skeletal muscle mass for diagnosing secondary sarcopenia in patients with malignancies, which should raise attention in SO diagnosis (66, 67).

No standard interventions or treatments for SO have been established. Comorbidity control, cardiopulmonary function promotion, anesthetic risk management, minimal inversive surgery techniques, and complication surveillance should be basic requirements. The critical point for sarcopenia recovery is early and continuous intervention, starting at preoperative and continuing into postoperative and long-term care (56, 68). Although the feasibility and efficacy of preoperative reversion of SO remains insufficient, early nutritional managements may benefit patients with SO. The combined supplements of high-quality amino acids, proteins, and vitamin D were demonstrated to be effective for rehabilitation of sarcopenia (69, 70). This strategy could be introduced during the perioperative term to benefit metabolic stress and attenuated muscle wasting (13). Specific formulas enriched with immunonutrients (arginine, omega-3-fatty acids, and ribonucleotides) may help promote immune response during perioperative period (71). Postoperative rehabilitation and long-term body composition management must be considered for cancer patients with SO. The epidemiological prevention and management of SO for the whole population should also be beneficial.

The limitations of our study should be considered when interpreting the reported findings. The included studies were mostly retrospective with small samples. The inconsistencies in SO diagnosis and prevalence could impact meta-analysis outcomes although we had conducted subgroup analyses to resolve the problems. The significant heterogeneity in the meta-analysis of major complications was not resolved in subgroup analyses, and random effect models were thus adopted. The number of studies on specific cancer types was limited in this meta-analysis. To select the NSO patients as the comparator could increase the complexity in interpreting findings considering the mix of “only obese” and “only sarcopenic” patients; the NN patients was thus the preferred comparator. Notably, the assessment of skeletal muscle function was emphasized for SO diagnosis by the ESPEN-EASO consensus (66), however, this item had hardly been conducted by the included studies. Regarding obesity diagnosis, the single cut-off of BMI (Table 2) could be defective because of the changed body compositions with age and gender. The age-adjusted and gender-specific cut-offs of fat mass proportion were thus recommended (66). Further studies should investigate the roles of SO in surgical oncology following the guidelines of ESPEN-EASO consensus. In addition, the SO cannot be the simple adduct of sarcopenia and obesity, the characteristics and management of SO patients warrant further investigation in scientific and clinical studies.

## Conclusion

This study confirmed the adverse impact of SO on perioperative complications and survival outcomes in gastrointestinal surgical oncology. Interventions aiming at SO have potentials to promote surgery benefits for gastrointestinal cancer patients. The existing evidence suggested the combination of sarcopenia and obesity rather than the ratio of fat mass to muscle mass as the definition of SO. The rigorous diagnosis cutoffs may help recognize the real SO and achieve satisfied clinical significance. Further studies should investigate the roles of SO following the guidelines of ESPEN-EASO consensus.

## Data availability statement

The original contributions presented in this study are included in the article/Supplementary Material, further inquiries can be directed to the corresponding author/s.

## Author contributions

PW, ZL, XL, FY, and MQ conducted the literature search, study selection and data extraction, and data analysis. GL, SW, YM, and HL managed software and figures. PW and MQ accessed and verified the underlying data. All authors contributed to the study design, data interpretation, writing and editing of the manuscript, had full access to all the data in the study, and had final responsibility for the decision to submit for publication.

## Conflict of interest

GL was employed by company China Aerospace Science and Industry Corporation. The remaining authors declare that the research was conducted in the absence of any commercial or financial relationships that could be construed as a potential conflict of interest.

## Publisher’s note

All claims expressed in this article are solely those of the authors and do not necessarily represent those of their affiliated organizations, or those of the publisher, the editors and the reviewers. Any product that may be evaluated in this article, or claim that may be made by its manufacturer, is not guaranteed or endorsed by the publisher.
