# Supplementary material for: Sarcopenic obesity and therapeutic outcomes in gastrointestinal surgical oncology: A meta-analysis
Source: Front Nutr. 2022 Jul 22;9:921817. doi: 10.3389/fnut.2022.921817 (PMC9355157; doi:10.3389/fnut.2022.921817)
Supplement: Supplementary file 1 [file Data_Sheet_1.PDF]

Supplemental Tables and Figures

Supplemental Table 1: pg. 2

Supplemental Table 2: pg. 3-4

Supplemental Table 3: pg. 5

Supplemental Table 4: pg. 6

Supplemental Fig. 1: pg. 7

Supplemental Fig. 2: pg. 8

Supplemental Fig. 3: pg. 9

Supplemental Table 1 The search strategy for the PubMed database (title and abstract) using search terms.

Set #	Search for	Results (September 10^{t} 2021)
#1	((((((sarcopenia[Title/Abstract]) OR (sarcopenic[Title/Abstract])) OR (myopenia[Title/Abstract])) OR (myopenic[Title/Abstract]))	11,616
#2	((((obese[Title/Abstract]) OR (obesity[Title/Abstract])) OR (adiposity[Title/Abstract])) OR (adipose[Title/Abstract])))	410,765
#3	((((cancer[Title/Abstract]) OR (tumor[Title/Abstract])) OR (carcinoma[Title/Abstract])) OR (oncology[Title/Abstract])))	2,940,785
#4	((((((surgical[Title/Abstract]) OR (surgery[Title/Abstract])) OR (operation[Title/Abstract])) OR (resection[Title/Abstract])) OR (ectomy[Title/Abstract])) OR (operative[Title/Abstract]))	2,402,560
#7	(((#1 AND #2) AND #3) AND #4)	138

Supplemental Table 2. List of excluded studies with reasons for exclusion

First author, year	Reasons	Reference
Ngo-Huang A, 2021	Lack of endpoints	Ngo-Huang A, Herbert A, Fontillas RC, Parker NH, Asumbrado R, Garg N, Dibaj S, Liu DD, Ng AH, Guo Y, Shin KY, Katz MHG, Bruera E. Frequency of Sarcopenia, Sarcopenic Obesity, and Changes in Physical Function in Surgical Oncology Patients Referred for Prehabilitation. Integr Cancer Ther. 2021 Jan-Dec;20:15347354211000118. doi: 10.1177/15347354211000118. PMID: 33829906; PMCID: PMC8040607.
Donkers H, 2021	Other cancers-endometrial cancer	Donkers H, Fasmer KE, Mcgrane J, Pijnenborg JMA, Bekkers R, Haldorsen IS, Galaal K. The role of sarcopenic obesity in high-grade endometrial cancer. Int J Gynaecol Obstet. 2021 Aug;154(2):248-255. doi: 10.1002/ijgo.13591. Epub 2021 Feb 26. PMID: 33445216.
Sugawara K, 2020	Lack of endpoints	Sugawara K, Yamashita H, Okumura Y, Yagi K, Aikou S, Seto Y. Age-dependent survival impact of body mass index in patients with oesophageal squamous cell carcinoma. Eur J Surg Oncol. 2020 Oct;46(10 Pt A):1948-1955. doi: 10.1016/j.ejso.2020.05.012. Epub 2020 Jun 24. PMID: 32654887.
Nakamura H, 2020	Other cancers-breast cancer	Nakamura H, Makiguchi T, Yamaguchi T, Fujii T, Shirabe K, Yokoo S. Impact of skeletal muscle mass on complications following expander breast reconstruction. J Plast Reconstr Aesthet Surg. 2020 Jul;73(7):1285-1291. doi: 10.1016/j.bjps.2020.02.006. Epub 2020 Feb 15. PMID: 32201325.
Best TD, 2020	Other cancers-lung cancer	Best TD, Mercaldo SF, Bryan DS, Marquardt JP, Wrobel MM, Bridge CP, Troschel FM, Javidan C, Chung JH, Muniappan A, Bhalla S, Meyers BF, Ferguson MK, Gaissert HA, Fintelmann FJ. Multilevel Body Composition Analysis on Chest Computed Tomography Predicts Hospital Length of Stay and Complications After Lobectomy for Lung Cancer: A Multicenter Study. Ann Surg. 2020 Jul 8. doi: 10.1097/SLA.0000000000004040. Epub ahead of print. PMID: 32773626.
Pecorelli N, 2017	Lack of endpoints	Pecorelli N, Capretti G, Sandini M, Damascelli A, Cristel G, De Cobelli F, Gianotti L, Zerbi A, Braga M. Impact of Sarcopenic Obesity on Failure to Rescue from Major Complications Following Pancreaticoduodenectomy for Cancer: Results from a Multicenter Study. Ann Surg Oncol. 2018 Jan;25(1):308-317. doi: 10.1245/s10434-017-6216-5. Epub 2017 Nov 7. PMID: 29116490.
Tsaousi G, 2017	Non-standard assessment of muscle mass	Tsaousi G, Kokkota S, Papakostas P, Stavrou G, Doumaki E, Kotzampassi K. Body composition analysis for discrimination of prolonged hospital stay in colorectal cancer surgery patients. Eur J Cancer Care (Engl). 2017 Nov;26(6). doi: 10.1111/ecc.12491. Epub 2016 Mar 16. PMID: 26990464.
Lou N, 2017	Overlapping	Lou N, Chi CH, Chen XD, Zhou CJ, Wang SL, Zhuang CL, Shen X. Sarcopenia in overweight and obese patients is a predictive factor for postoperative complication in gastric cancer: A prospective study. Eur J Surg Oncol. 2017 Jan;43(1):188-195. doi: 10.1016/j.ejso.2016.09.006. Epub 2016 Sep 17. PMID: 27692536.
Batsis JA, 2016	Lack of endpoints	Batsis JA, Mackenzie TA, Jones JD, Lopez-Jimenez F, Bartels SJ. Sarcopenia, sarcopenic obesity and inflammation: Results from the 1999-2004 National Health and Nutrition Examination Survey. Clin Nutr. 2016 Dec;35(6):1472-1483. doi: 10.1016/j.clnu.2016.03.028. Epub 2016 Apr 7. PMID: 27091774; PMCID: PMC6432912.
Itoh S, 2016	Transplantation	Itoh S, Yoshizumi T, Kimura K, Okabe H, Harimoto N, Ikegami T, Uchiyama H, Shirabe K, Nishie A, Maehara Y. Effect of Sarcopenic Obesity on Outcomes of Living-Donor Liver Transplantation for Hepatocellular Carcinoma. Anticancer Res. 2016 Jun;36(6):3029-34. PMID: 27272822.
Anandavadivelan P, 2016	Lack of endpoints	Anandavadivelan P, Brismar TB, Nilsson M, Johar AM, Martin L. Sarcopenic obesity: A probable risk factor for dose limiting toxicity during neo-adjuvant chemotherapy in oesophageal cancer patients. Clin Nutr. 2016 Jun;35(3):724-30. doi: 10.1016/j.clnu.2015.05.011. Epub 2015 May 27. PMID: 26065721.

Lodewick TM, 2015	Lack of endpoints	Lodewick TM, Roeth AA, Olde Damink SW, Alizai PH, van Dam RM, Gassler N, Schneider M, Dello SA, Schmeding M, Dejong CH, Neumann UP. Sarcopenia, obesity and sarcopenic obesity: effects on liver function and volume in patients scheduled for major liver resection. J Cachexia Sarcopenia Muscle. 2015 Jun;6(2):155-63. doi: 10.1002/jcsm.12018. Epub 2015 Apr 28. PMID: 26136191; PMCID: PMC4458081.
Kuroki LM, 2015	Other cancers-endometrial cancer	Kuroki LM, Mangano M, Allsworth JE, Menias CO, Massad LS, Powell MA, Mutch DG, Thaker PH. Pre-operative assessment of muscle mass to predict surgical complications and prognosis in patients with endometrial cancer. Ann Surg Oncol. 2015 Mar;22(3):972-9. doi: 10.1245/s10434-014-4040-8. Epub 2014 Sep 5. PMID: 25190123; PMCID: PMC4355998.
Cooper AB, 2015	Enrolment of patients without surgery	Cooper AB, Slack R, Fogelman D, Holmes HM, Petzel M, Parker N, Balachandran A, Garg N, Ngo-Huang A, Varadhachary G, Evans DB, Lee JE, Aloia T, Conrad C, Vauthey JN, Fleming JB, Katz MH. Characterization of Anthropometric Changes that Occur During Neoadjuvant Therapy for Potentially Resectable Pancreatic Cancer. Ann Surg Oncol. 2015 Jul;22(7):2416-23. doi: 10.1245/s10434-014-4285-2. Epub 2014 Dec 18. PMID: 25519927.

Supplemental Table 3 Quality assessment of included studies according to the Newcastle-Ottawa Scale (NOS) for cohort/case-control studies.

Study, year	Selection				Comparability	Outcome			Total score
	Representativeness of the exposed cohort	Selection of the non-exposed cohort	Ascertainment of exposure	Demonstration that outcome of interest was not present at start of study	Comparability of cohorts on the basis of the design or analysis	Assessment of outcome	Was follow-up long enough for outcomes to occur	Adequacy of follow up of cohorts	
Rodrigues 2021	★	★	★	☆	★★	★	★	★	8
Peng 2021	★	★	★	☆	★☆	★	★	★	7
Olmez 2021	★	★	★	☆	☆☆	★	★	★	6
Kim 2021	★	★	★	☆	★☆	★	★	★	7
Fehrenbach 2021	★	★	★	☆	☆☆	★	★	★	6
Pedrazzani 2020	★	★	★	☆	★★	★	★	★	8
Onishi 2020	★	★	★	☆	★★	★	★	★	8
Han 2020	★	★	★	☆	★☆	★	★	★	7
Giani 2020	★	★	★	☆	☆☆	★	★	★	6
Kroh 2020	★	★	★	☆	★★	★	★	★	8
Kobayashi 2019	★	★	★	☆	★★	★	★	★	8
Jang 2019	★	★	★	☆	★☆	★	★	★	7
Gruber 2019	★	★	★	☆	★★	★	★	★	8
Berkel 2019	★	★	★	☆	★☆	★	★	★	7
Zhang 2018	★	★	★	★	★★	★	★	★	9
Martin 2018	★	★	★	☆	★☆	★	★	★	7
Chen 2018	★	★	★	★	★☆	★	★	★	8
Okumura 2017	★	★	★	☆	★☆	★	★	★	7
Sandini 2016	★	★	★	☆	★☆	★	★	★	7
Pecorelli 2016	★	★	★	☆	★☆	★	★	★	7
Nishigori 2016	★	★	★	☆	★☆	★	★	★	7
Malietzis 2016	★	★	★	☆	★☆	★	★	★	7
Grotenhuis 2016	★	★	★	☆	☆☆	★	★	★	6
Boer 2016	★	★	★	☆	☆☆	★	★	★	6
Lodewick 2015	★	★	★	☆	★★	★	★	★	8
Peng 2015	★	★	★	☆	☆☆	★	★	★	6

A score of 5 or below was considered low quality; a score of 6 or 7 was considered moderate quality; and a score of 8 or 9 was considered high quality.

Supplemental Table 4 Meta-analyses of clinical characteristics of sarcopenia obesity patients.

Parameter	Comparisons	Study and Sample	Meta-analysis		Heterogeneity [a]		Publication bias		Sensitivity analysis
			WMD/*OR* (95% CI)	*P* value	I^2 (%)	P	Funnel plot	Egger's test	
Age, years	SO vs. NN	1160 [14, 17, 24, 28, 30, 34]	8.65 (6.24-11.05)	<0.001	63.7	0.017	Negative	0.50	Negative
	SO vs. NSO	1018 [19, 20, 23, 31, 32, 38]	4.02 (-0.08-8.13)	0.055	89.6	<0.001	Negative	0.24	Negative
Gender (male)	SO vs. NN	1160 [14, 17, 24, 28, 30, 34]	*2.63 (0.39-17.9)*	0.32	94.5	<0.001	Positive	0.080	Negative
	SO vs. NSO	4107 [14, 17, 19, 20, 23-26, 28, 30-32, 34, 38]	*1.49 (0.85-2.60)*	0.16	85.1	<0.001	Negative	0.18	Negative
ASA grades (3-4)	SO vs. NN	945 [14, 17, 28, 30, 34]	*2.99 (1.99-4.49)*	<0.001	0	0.95	Negative	0.09	Negative
	SO vs. NSO	2709 [14, 17, 19, 23, 28, 30, 34, 38]	*1.64 (1.26-2.15)*	<0.001	0	0.51	Negative	0.86	Negative
Cancer stage (III-IV)	SO vs. NN	1160 [14, 17, 24, 28, 30, 34]	*1.28 (0.93-1.77)*	0.13	10.6	0.35	Negative	0.26	Negative
	SO vs. NSO	3136 [14, 17, 19, 23, 24, 26, 28, 30, 34]	*0.99 (0.66-1.49)*	0.98	63.1	0.006	Negative	0.28	Negative

[a] Once high heterogeneity was confirmed (I^2>50% or P<0.05), a random-effects model was used, otherwise, a fixed-effects model was used.

ASA: American Society of Anesthesiology; CI, confidence interval; NN: non-sarcopenia, non-obesity; NSO, non-sarcopenia obesity; OR, odds ratio; SO sarcopenia obesity; WMD, weighted mean difference;.

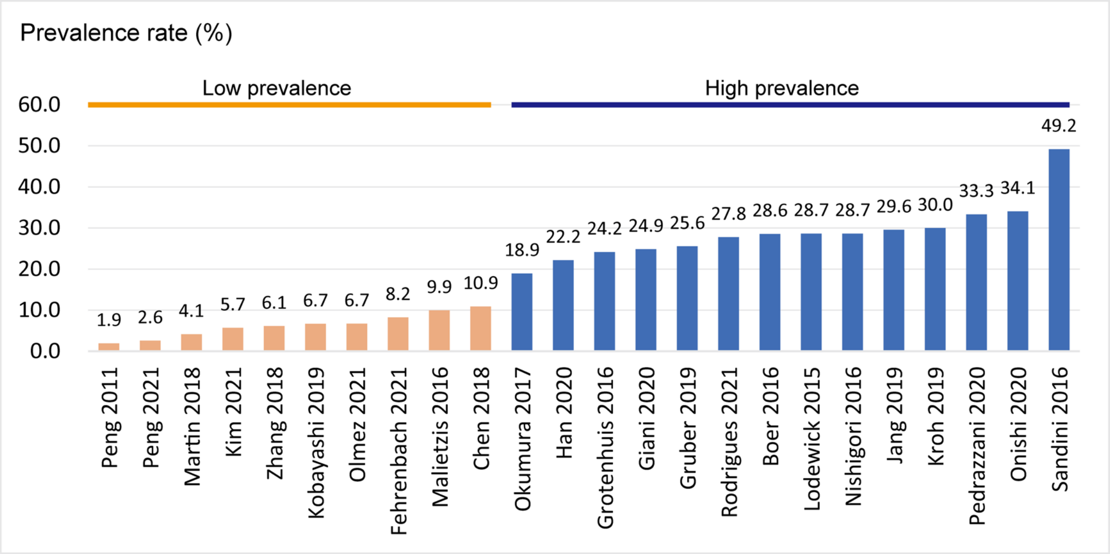

Supplemental-Fig. 1 Distributions of reported prevalence rate of sarcopenia obesity among included studies. Studies are divided into low prevalence group (<10%) and high prevalence group (>20%).

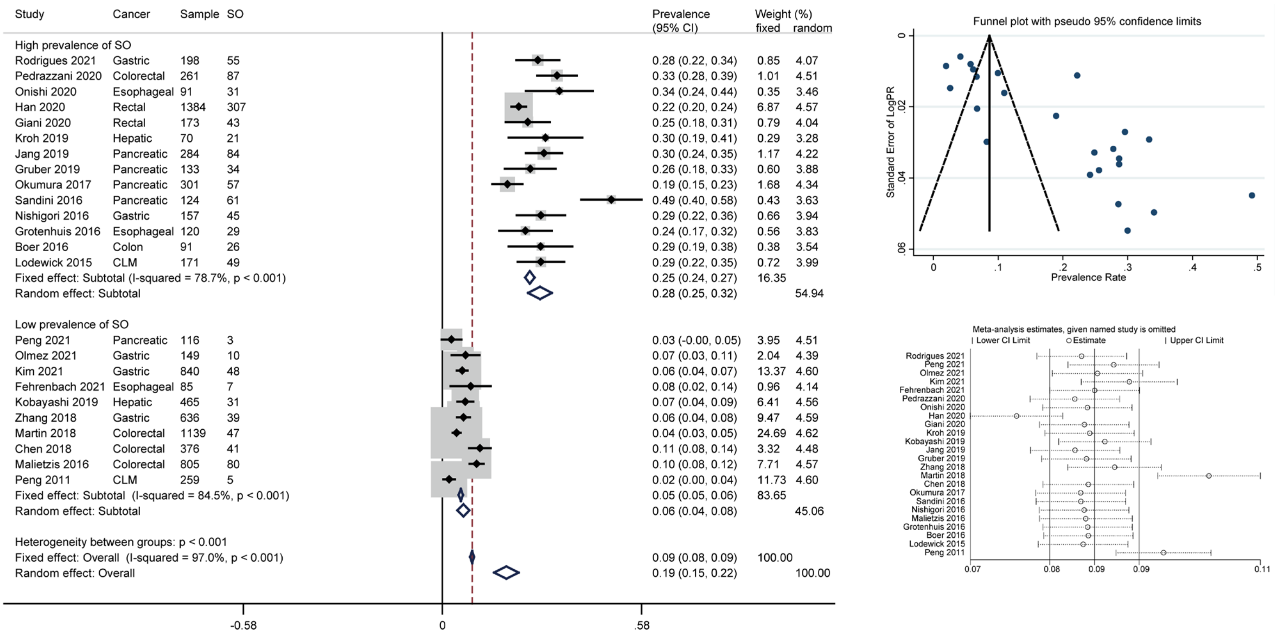

Supplemental -Fig. 2 Forest plot of meta-analysis for prevalence rates of sarcopenia obesity. The funnel plot and sensitivity analysis are provided. CI: confidence interval; CLM: colorectal liver metastasis; SO: sarcopenia obesity.

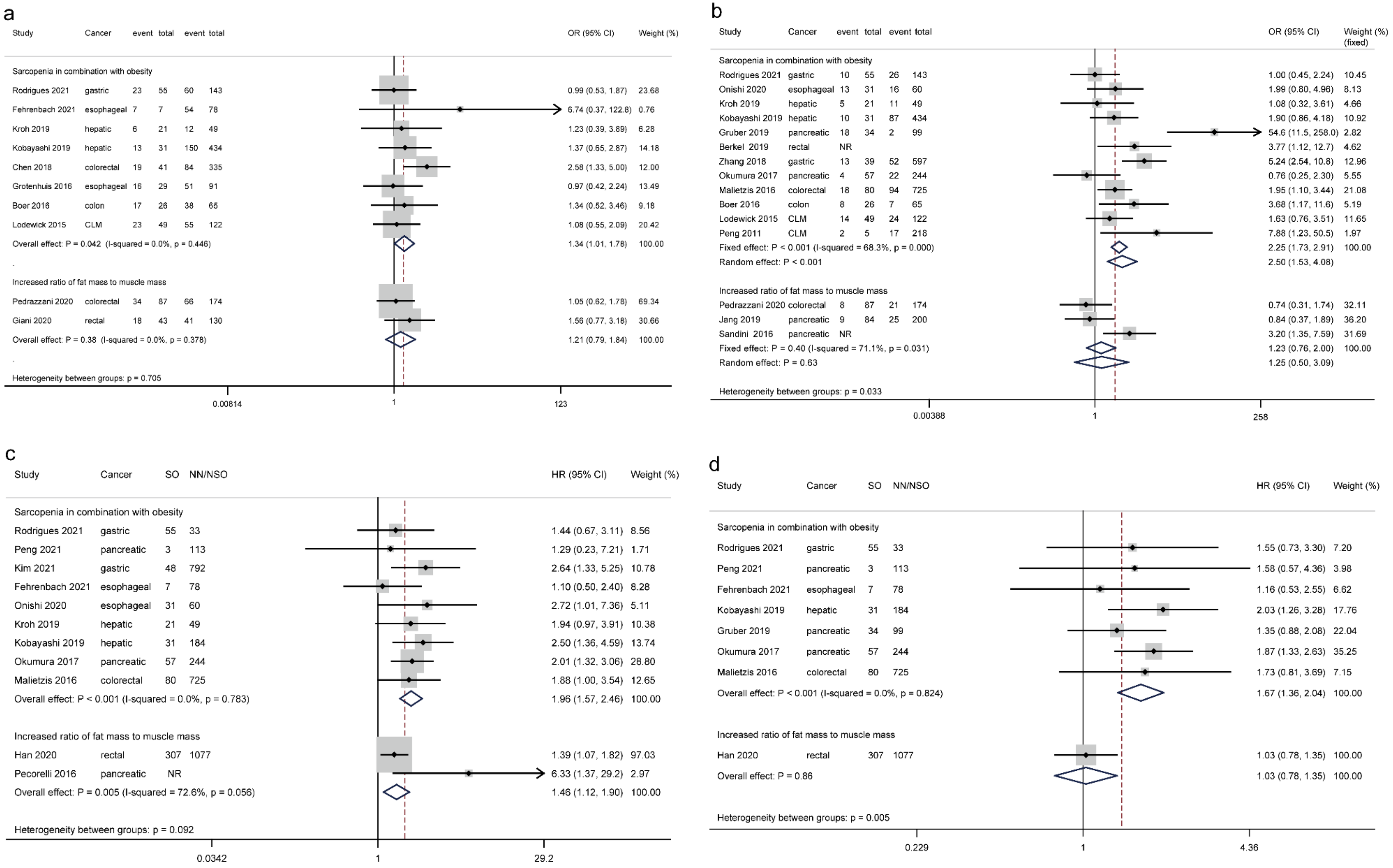

Supplemental -Fig. 3 Forest plots of subgroup analyses for sarcopenia obesity and primary outcomes stratified by definitions of sarcopenia obesity. **a:** Total complications; **b:** Major complications; **c:** Overall survival; **d:** Disease-free survival. CI: confidence interval; CLM: colorectal liver metastasis; HR: hazard ratio; NN: non-sarcopenia non-obesity; NR: not reported; NSO: non-sarcopenia obesity; OR, odds ratio; SO: sarcopenia obesity.
